# Endometrial cancer in a renal transplant recipient: A case report

**DOI:** 10.1515/med-2020-0118

**Published:** 2020-10-01

**Authors:** Na Liu, Lei Yang, Yan Long, Guoqing Jiang

**Affiliations:** Department of Gynecology & Obstetrics, Beijing Friendship Hospital, Capital Medical University, No. 95, Yong’an Road, Beijing 100050, China

**Keywords:** renal transplantation, endometrial carcinoma, chemotherapy, case report

## Abstract

As the most effective treatment for end-stage renal diseases, renal transplantation can improve the quality of life of patients and prolong the survival time. However, during the prolonged survival time, malignancy has become one of the main causes of death of recipients, which vary geographically. Tumors in the digestive system and urothelial tumors have been highly reported in Asia. In general, the gynecological malignant tumors have been rarely reported, especially the endometrial carcinoma. Herein, a 63-year-old female renal transplant recipient diagnosed with endometrial carcinoma (15 years after transplantation) was reported. The patient had suffered irregular postmenopausal bleeding for a short time before hospitalization. She underwent abdominal hysterectomy, bilateral salpingo-oophorectomy, right pelvic lymphadenectomy, right para-aortic lymphadenectomy and omental excision. Postoperative pathology showed ovarian and pelvic lymph node metastasis and pathological stage IIIC. After six courses of chemotherapy with paclitaxel 270 mg + carboplatin 500 mg, the patient’s renal function was normal. During the third cycle of chemotherapy, the patient suffered a third-degree bone marrow suppression and returned to normal soon when treated with the recombinant human granulocyte stimulating factor. In conclusion, early screening of gynecologic tumors is important for female patients after renal transplantation, which has a positive significance for the prognosis improvement.

## 1 Introduction

As the most effective treatment for end-stage renal failure, renal transplantation can improve the survival of patients and their quality of life. However, with prolonged survival time and the application of immunosuppressive agents, malignancies might become one of the main causes of death for renal transplant recipients with functioning graft.

There have been reports concerning skin cancer and lymphoma in renal transplant recipients [1], and one study from our hospital has revealed that urothelial cancer is the predominant tumor in Chinese renal transplant recipients [2]. However, there are few reports about the gynecological malignancies except for cervical cancer in renal transplant recipients.

In the United States, endometrial cancer is the fourth most common invasive gynecologic cancer [3]. Among the renal transplant recipients, endometrial cancer is sporadic, and the standardized incidence ratio for uterine cancers has been reported to range from 0.9 to 2.1 per 1,00,000 females [4,5,6,7,8,9,10,11,12]. In this study, an endometrial cancer patient with renal allograft was reported, and cisplatin-based chemotherapy in renal transplant recipients was also explored. The importance of a close follow-up after renal transplant surgery was highlighted, including regular gynecologic screening for endometrial lesions.

## 2 Case report

A 63-year-old patient (gravida 2, and para1) was admitted to the Beijing Friendship Hospital for postmenopausal bleeding, with the diagnosis of uterus endometrial lesions. The patient had received left kidney transplantation without bilateral nephrectomy in 2002 because of the end-stage renal disease secondary to chronic glomerulonephritis. Then, she menopaused in 2004. At admission, during the 16 years following the renal transplantation, the patient had been on cyclosporine A (150 mg twice daily), prednisone (60 mg once daily), and mycophenolate mofetil (1,500 mg twice daily). During the perimenopause period, the patient was subjected to the ultrasound examination of uterus, and no abnormality had been detected.

**Figure 1:**
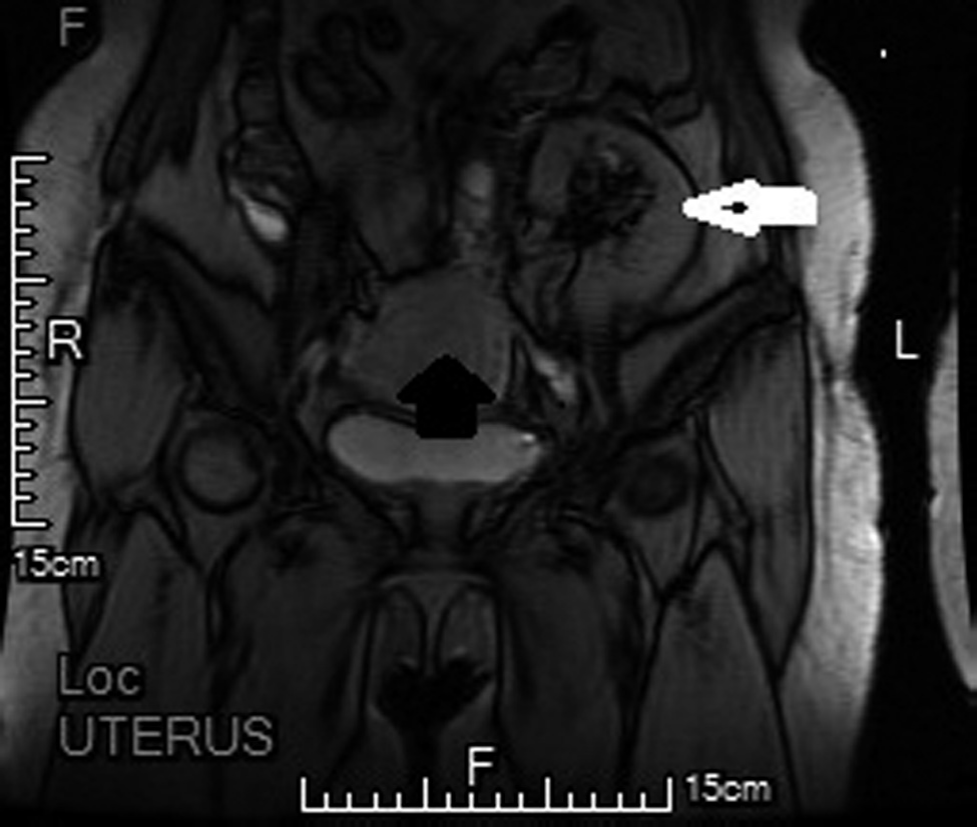
Preoperative MRI image. The white arrow indicates the transplanted kidney. The black arrow indicates the FIGO-stage IA endometrial carcinoma.

**Figure 2:**
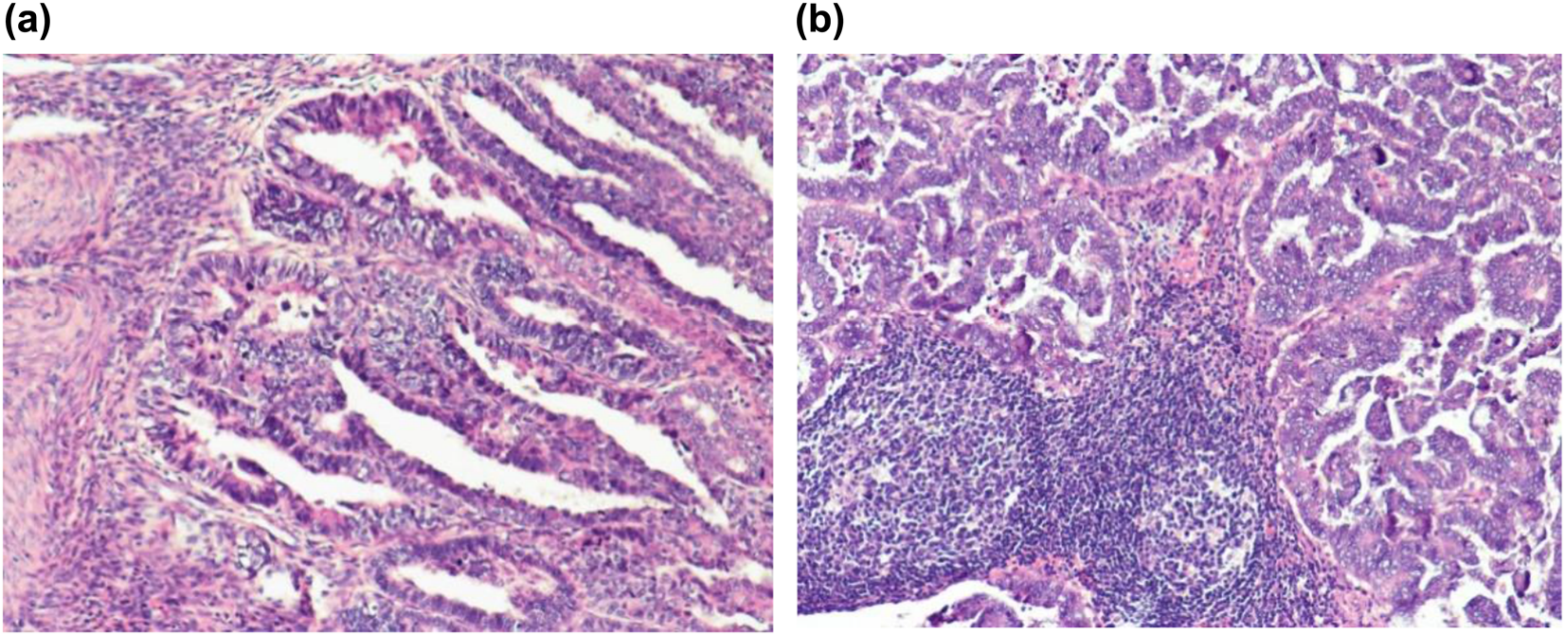
Postoperative pathology images. (a) HE staining of the uterus after surgery showed that the tumor invaded more than half of the muscle layer, and some reached the serosa layer. Magnification: 100×. (b) Lymph node metastasis after the tumor surgery, shown by HE staining. Magnification: 100×.

The patient complained of vaginal bleeding for more than 10 days before the hospitalization. Clinical laboratory test results of tumor biomarkers showed that pre-operative CA125 was 332.5 U/mL and pre-operative CA199 was 111.14 U/mL. Transvaginal ultrasound revealed that the normal size and shape of uterus and the anteverted endometrium (8 mm anteriorly, 9 mm posteriorly) with fluid in cavity. The total endometrial thickness was 17 mm, with rich blood flow. Endometrial cancer was confirmed after the hysteroscopy and segmental curettage. Pelvic magnetic resonance imaging (MRI) indicated the possibility of the FIGO-stage IA endometrial carcinoma (Figure 1). The patient underwent abdominal hysterectomy, bilateral salpingo-oophorectomy, right pelvic lymphadenectomy, right para-aortic lymphadenectomy and omental excision. Pathology report confirmed the FIGO-stage IIIC endometrial adenocarcinoma with extrailiac lymph node metastasis (Figure 2). Both tumor biomarkers of CA125 and CA199 returned to normal after operation (less than 30 µ/mL).

The patient started the adjuvant treatment of carboplatin (500 mg) and paclitaxel (270 mg), starting from day 7 after surgery. At the first follow-up during the three cycles of chemotherapy, the patient complained of sciatic nerve pain that had been worsening in the past several weeks. Moreover, after the third cycle of chemotherapy, serious marrow suppression occurred, without liver or kidney injuries, which recovered soon after the application of G-CSF. The sixth cycle of chemotherapy was completed in July 2017. Moreover, she was subjected to immunosuppressive therapy during and after chemotherapy as before treatment. Thereafter, the patient was closely followed up in the outpatient clinic. The renal function indicator during surgery and chemotherapy showed that creatinine fluctuated at 60–92 µmol/L (within the normal range). The glomerular filtration rate was 116 mL/min before chemotherapy and 122 mL/min after chemotherapy. No recurrence was observed according to the computed tomography scanning at 28 months after surgery.

**Ethical statement:** This study was approved by Beijing Friendship Hospital, Capital Medical University. Written informed consent was obtained from this patient.

## 3 Discussion

Malignancies represent one of the main causes of death for renal transplant recipients with prolonged survival time, with other influencing factors such as the ages of donor and recipient and the responses of immunosuppressive therapy. Studies have suggested that malignant tumors would occur in about 20% of patients within 10 years after renal transplantation [13]. Malignancies after renal transplantation exhibited significant differences in different endemic and geographic regions. Skin cancer and lymphoma are most common in Europe (40%) and the USA (12–30%) [4], while digestive tract tumors are most common in Japan (57%) and South Korea (40%) [14,15]. Moreover, cervical and vulvovaginal tumors in renal transplant recipients have been reported approximately five times more common in the US population [16]. Bobrowska et al. [17] reported that endometrial hyperplasia was detected in 31 cases (69%) of the renal allograft recipients and that endometrial cancer was only detected in one case (2%) of the renal allograft recipients. Although women receiving renal transplant seem to have extremely high risk of endometrial hyperplasia, there are rare reports of endometrial cancer in the renal transplant recipients. There is no previous article focusing on the treatment of endometrial cancer in the renal transplant recipients. In this paper, we first discussed the treatment, especially chemotherapy, immunosuppressive program of the endometrial cancer in the renal transplant recipients.

In this study, the renal transplant patient had postmenopausal vaginal bleeding for more than 10 days. Postoperative pathology results confirmed stage IIIC cancer. The main risk factors for endometrial cancers are related to excessive estrogen, nulliparity, diabetes mellitus, and tamoxifen application. Estrogen stimulation has been believed to be the main etiologic factor for endometrial cancer development [18]. Cancers of renal transplant recipients are always characterized by enhanced aggressiveness, as compared with patients receiving no transplantation. Effects of immunosuppressive drugs related to aggressiveness should be taken into account.

There would be alterations in the anatomic structure of the pelvis after the transplantation of a recipient kidney, such as the adhesions of the kidney with surrounding tissues, and connection of the renal arteriovenous with iliac vessels. It is difficult to perform ipsilateral pelvic lymphadenectomy without inducing any injuries for the donor kidney. In the case reported herein, the transplanted kidney was firmly adherent to the iliac vessels, which totally obliterated the obturator fossa. Therefore, no pelvic lymphadenectomy was performed on the left side because of the risks concerning renal function loss and fatal vascular injury. For patients with advanced tumors, abdominal aortic lymphadenectomy would be necessary. Although the para-aortic tissue may adhere to the transplanted kidney, it is still possible to remove the adhesive para-aortic lymph nodes. The patient reported herein was associated with advanced endometrial cancer and underwent a successful para-aortic lymphadenectomy. It was noteworthy that complete hemostasis in the surgical area played a key role because of the serious adhesion.

Renal transplant recipients with gynecological malignant tumors have been rarely reported. Therefore, the experience of chemotherapy treatment could only be learned from other malignant tumors. The combination of chemotherapeutic agents and immunosuppressive agents often leads to severe bone marrow suppression. Taxanes exert antineoplastic activity by stabilizing cell microtubules, which thus inhibits cellular mitosis and lead to cell cycle arrest. Common toxic effects mainly include myelosuppression, nausea and vomiting, and hypersensitivity reactions. Given this extensive liver metabolism, dose reductions are necessary with hepatic dysfunction. Paclitaxel can be safely used in patients, even in those with renal insufficiencies [19]. The platinum agents would induce the impairment of DNA replication and transcription, therefore resulting in apoptosis. The major toxic effects of cisplatin and carboplatin include myelosuppression, nephrotoxicity, neurotoxicity and ototoxicity [20]. Given the high propensity of nephrotoxicity, cisplatin is contraindicated in preexisting renal impairment. Dose reductions are thought to be necessary for renal function in some cases [2]. In the case reported herein, the patient was treated with a paclitaxel plus carboplatin regimen, every 4 weeks, for a total of six courses (paclitaxel 175 mg/m^2^, and carboplatin 300 mg/m^2^; all of them were regular doses). Severe myelosuppression occurred after the third course, which recovered soon after the application of the granulocyte-stimulating factor, with normal renal function. Of course, chemotherapy for the transplant recipients still needs to be further explored in future.

Specific recommendations for modifications of immunosuppression following malignancy diagnosis in transplant recipients have not been well established. The most common approach is the reduction or even elimination of maintaining immunosuppression [21]. Without modifications of immunosuppression drug dose, the patient in this study had normal renal function during the treatment.

In order to avoid pelvic graft injury, no radiotherapy was conducted. There are seldom reports of postoperative radiotherapy for malignancies in renal transplant recipients. However, Nicola et al. [22] have reported a case of the application of intensity-modulated radiation therapy (IMRT) for a 60-year-old man with T3bN1M0 prostate adenocarcinoma. Their results have shown that complex dose distributions achieved with IMRT enable the safe delivery of radical dose-escalated radiation therapy to the pelvic lymph nodes in the presence of a pelvic-transplanted kidney. Renal transplant should not be considered as a contraindication to pelvic radiotherapy.

In summary, gynecologic tumors after kidney transplantation have been rarely reported. Radical excision of the lesion should be considered based on the evaluation of the graft function. Moreover, radiotherapy and chemotherapy are feasible. Early screening for gynecological tumors after kidney transplantation is particularly important, which can significantly improve the disease prognosis.
